# Viral Genome-Linked Protein (VPg) Is Essential for Translation Initiation of Rabbit Hemorrhagic Disease Virus (RHDV)

**DOI:** 10.1371/journal.pone.0143467

**Published:** 2015-11-23

**Authors:** Jie Zhu, Binbin Wang, Qiuhong Miao, Yonggui Tan, Chuanfeng Li, Zongyan Chen, Huimin Guo, Guangqing Liu

**Affiliations:** Shanghai Veterinary Research Institute, Chinese Academy of Agricultural Sciences (CAAS), Shanghai, 200241, China; University of British Columbia, CANADA

## Abstract

Rabbit hemorrhagic disease virus (RHDV), the causative agent of rabbit hemorrhagic disease, is an important member of the *caliciviridae* family. Currently, no suitable tissue culture system is available for proliferating RHDV, limiting the study of the pathogenesis of RHDV. In addition, the mechanisms underlying RHDV translation and replication are largely unknown compared with other *caliciviridae* viruses. The RHDV replicon recently constructed in our laboratory provides an appropriate model to study the pathogenesis of RHDV without *in vitro* RHDV propagation and culture. Using this RHDV replicon, we demonstrated that the viral genome-linked protein (VPg) is essential for RHDV translation in RK-13 cells for the first time. In addition, we showed that VPg interacts with eukaryotic initiation factor 4E (eIF4E) *in vivo* and *in vitro* and that eIF4E silencing inhibits RHDV translation, suggesting the interaction between VPg and eIF4E is involved in RHDV translation. Our results support the hypothesis that VPg serves as a novel cap substitute during the initiation of RHDV translation.

## Introduction

**Fig 1:**
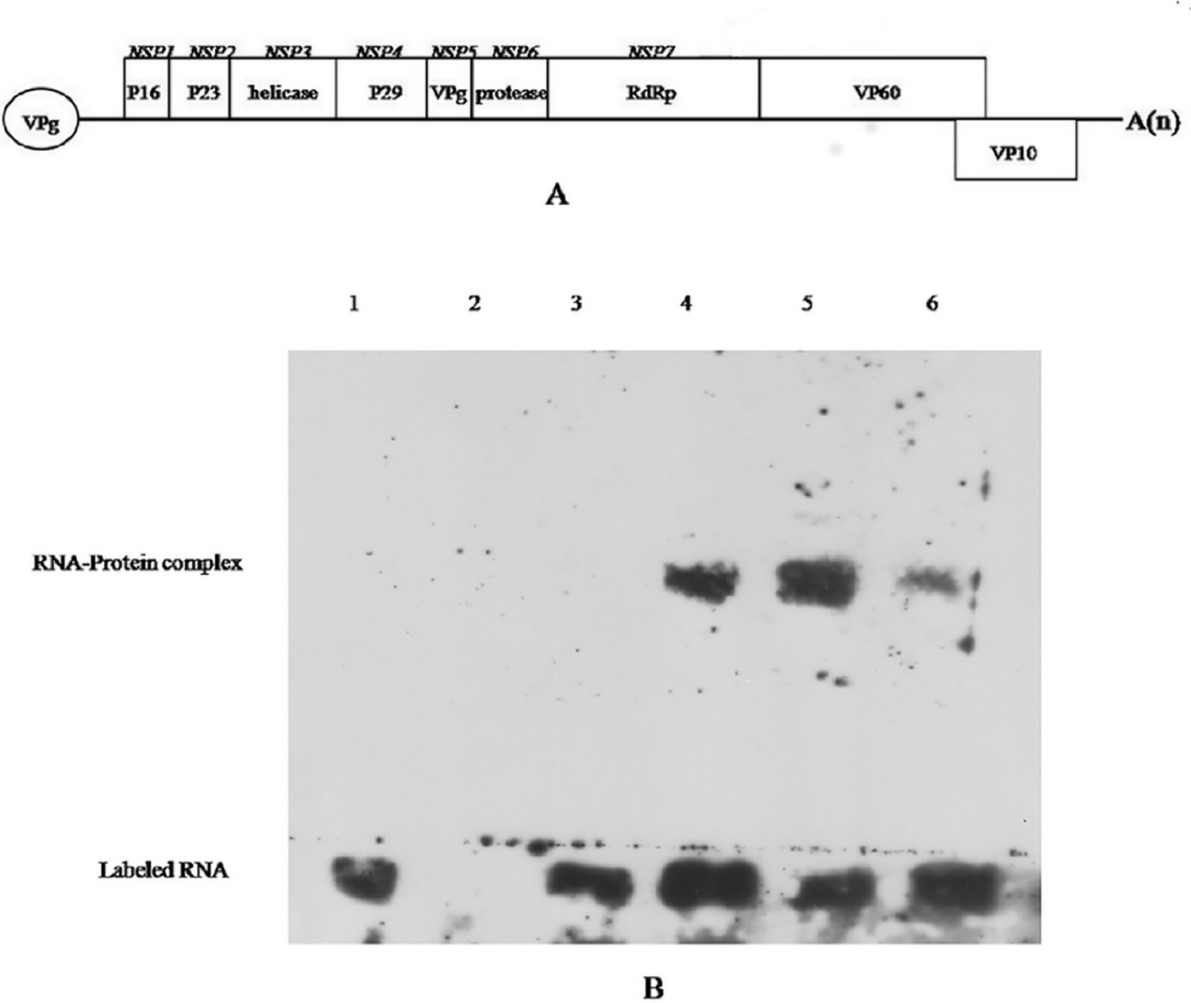
The RHDV genome is associated with the fully processed viral protein genome (VPg) at its terminus. (A) A schematic diagram of the RHDV genome showing the genes encoding non-structural (NSP1-NSP7) and structural (VP60 and VP10) proteins. (B) The VPg protein interacted with RHDV 5′ -Extreme RNA, as determined by the RNA EMSA assay. Lane 1: negative control: VPg protein (2 μg) + labeled RHDV-NSP2 (6 nM); Lane 2: blank control: VPg protein (2 μg); Lane 3: negative control: labeled 5′ -Extreme RNA (6 nM); Lane 4: VPg protein (1 μg) + labeled 5′ -Extreme RNA (6 nM); Lane 5: VPg protein (2 μg) + labeled 5′ -Extreme RNA (6 nM); Lane 6: competition group: VPg protein (2 μg) + 5′ -Extreme RNA (6 nM) + labeled 5′ -Extreme RNA (6 nM).

Rabbit hemorrhagic disease virus (RHDV) is the causative agent of rabbit hemorrhagic disease (RHD), which is characterized by hemorrhaging, liver necrosis, and high mortality [1]. After its initial identification in China in 1984 [2], RHD outbreaks have subsequently been reported in other Asian countries [3], Europe [4], Mexico [5], and other countries worldwide [6]. The RHDV belonging to the *Caliciviridae* family [1, 7, 8] is a positive-sense, single-stranded RNA virus. The complete genomic sequence of a German isolate of RHDV has been determined and is 7,437 nucleotides (nt) in length [7]. A long open reading frame (ORF) of 2,344 codons (ORF1) and a short ORF of 118 codons (ORF2) have been identified in the genome of RHDV. A nonstructural protein (NSP1-7) and virion coat protein (VP60) were predicted in the C-terminus of ORF1, and a minor structural protein (VP10) was identified in the N-terminus of ORF2 [7, 9]. The 5′ end of the RHDV genome is covalently linked to the viral protein, VPg [10], and a polyadenylated tail was identified at the 3′ end of the RHDV genome, as shown in Fig 1A. Due to the lack of a suitable culture system for RHDV, the mechanism of RHDV translation is largely unknown compared with other RNA viruses, such as foot-and-mouth disease virus, avian influenza virus, and poliovirus [11]. However, Goodfellow *et al*. have significantly improved our understanding of the translation of calicivirus mRNA. Their results demonstrated that caliciviruses use a novel protein-directed translation mechanism in which the interaction between translation initiation factors and VPg plays an important role [12–14]. This novel translation mechanism has never been reported in any other animal RNA viruses. While the virus-encoded protein (VPg) is covalently linked to the 5′ end of the RHDV genome [10], the biological functions of VPg, especially its role in translation, are not clear. In the present study, we provide evidence that VPg plays an essential role in RHDV translation. Deletion of VPg significantly inhibited RHDV translation, and this effect was restored by trans-supplementation of VPg protein. Additionally, VPg interacted with eukaryotic initiation factor 4E (eIF4E), and eIF4E silencing significantly inhibited RHDV translation. Our results suggested that VPg serves as a novel ‘cap substitute’ during the initiation of RHDV mRNA translation.

## Materials and Methods

### RHDV strain, RK-13 cells, and preparation of viral cDNA

The RHDV strain CHA/JX/97 was obtained from a rabbit in Zhejiang (China) during the 1997 outbreak. The genomic sequence of RHDV CHA/JX/97 is available in GenBank through accession number DQ205345. The viral cDNA was generated according to our previous report [15]. RK-13 cells (ATCC NO: CCL-37) were grown in 5% CO_2_ at 37°C in minimal essential medium (MEM) (HyClone, USA) containing 10% fetal bovine serum (FBS) (Gibco, USA).

### Plasmid construction and transfection

The pRHDV-luc plasmid, in which the *VP60* and partial *VP10* genes were replaced with the Fluc gene, and the VPg-deletion mutant of the pRHDV-luc (pRHDV-luc/ΔVPg) plasmids were generated in our previous study [16]. The pTVT-RHDV plasmid containing the complete RHDV cDNA and the T7 promoter and ribozyme, was generated in our previous study [17]. The pVPg plasmid, which encodes RHDV VPg, was constructed by inserting the VPg cDNA into a pcDNA3.1/Zeo (+) vector (Invitrogen, USA) using K*pn*I and E*coR*I. The VPg cDNA was cloned into the pACT vector (Promega, USA) using X*ba*I and K*pn*I to generate the pACT-VPg plasmid. Similarly, the rabbit eIF4E cDNA was inserted into the pBIND vector (Promega, USA) using X*ba*I and K*pn*I to generate the pBIND-eIF4E plasmid. A pV5-VPg plasmid, which encodes RHDV VPg and a V5 label, was constructed by cloning the VPg cDNA into a pEASY-M1 Expression Vector (Trans Gen Biotech, China). The *eIF4E* gene was subcloned into the pEASY-M2 Expression Vector (Trans Gen Biotech, China) to generate the pMyc-eIF4E plasmid with a Myc label. The VPg cDNA was inserted into the pBiFC-VN155 (I152L) vector (Addgene plasmid #27097) [18] using E*coR*I and K*pn*I to generate the pVPg-VN plasmid. The *eIF4E* gene was subcloned into the pBiFC-VC155 vector (Addgene plasmid #22011) [19] to generate the peIF4E-VC plasmid. The plasmids pbJunVN155 (Addgene plasmid #27098), pbFosVC155 (Addgene plasmid #22013) and pbFos(ΔZIP)VC155 (Addgene plasmid #22014) were purchased from Addgene [19]. The p4E-BP1 plasmid, which encodes eIF4E-binding protein (4E-BP1), was constructed by cloning the 4E-BP1 cDNA into a pcDNA3.1/Zeo(+) vector (Invitrogen, USA) using B*amH*I and E*coR*I. The pIRES-Rluc plasmid containing the internal ribosome entry site (IRES) from encephalomyocarditis virus (EMCV) was constructed by inserting the Renilla luciferase (Rluc) cDNA into a pIRES vector (Clontech, USA) using X*ba*I and N*ot*I. The peGFP plasmid was constructed by cloning the eGFP cDNA into a pcDNA3.1/Zeo(+) vector (Invitrogen, USA) using B*amH*I and E*coR*I.

RK13 cells or 293T cells were cultured in 6-, 12- or 24-well plates (NEST) at 37°C in a humidified incubator with 5% CO_2_. The X-tremeGENE HP DNA transfection reagent (Roche, Switzerland) was used to transfect cells using the plasmids mentioned above according to the manufacturer’s instructions.

### Evaluation of the interaction between VPg protein and the 5′ -Extreme RNA of RHDV

The 5′ -Extreme RNA of RHDV, including the 5′ UTR and NSP1-coding region of RHDV, was produced by *in vitro* transcription using the T7 RNA polymerase RiboMAX Large Scale RNA Production System (Promega, USA). A PCR assay was used to generate the 5′ -Extreme RHDV fragment. The primers used were RHDV-T7-F (5′-CGAAATTAATACGACTCATAT-3′) and NSP1-R (5′-TTCAAAAACAGAGGGGGAAGA-3′), and the pTVT-RHDV plasmid served as the template. RHDV-NSP2 was used as an irrelevant PCR control with primers NSP2-F (5′-GGGGAAGTTGACGACCTGTTT-3′) and NSP2-R (5′-CTCAAACGTGTCAAACAACCT-3′) and the pTVT-RHDV plasmid as template. After transcription, a single biotinylated nucleotide was attached to the 3′ terminus of the 5′ -Extreme RNA of RHDV using the RNA 3′ End Biotinylation Kit (Pierce, USA). The interaction between VPg protein and the 5′ -Extreme RNA was evaluated using a LightShift Chemiluminescent RNA EMSA Kit (Pierce, USA) according to the manufacturer’s instructions. The unlabeled 5′ -Extreme RNA was used as a competition control. Biotinylated RNA was detected using the Chemiluminescent Nucleic Acid Detection Module Kit (Pierce, USA).

### Luciferase activity measurements

Twenty-four hours after transfection, the RK13 cells were washed with PBS and lysed in 200 μl of Passive Lysis Buffer (Promega, USA). After gentle shaking for 15 min at room temperature, the cell lysate was transferred to a tube and centrifuged for 2 min at 12,000 ×g and 4°C. The supernatant (20 μl) was added to 100 μl of luciferase assay substrate to evaluate the activity of firefly luciferase (Fluc) and Renilla luciferase (Rluc) using a dual-luciferase reporter assay system (Promega, USA) based on relative light units (RLUs). The luciferase activities were analyzed using a FB12 Luminometer (Berthold, Germany). To normalize the luciferase values determined for cells transfected with the firefly luciferase replicon, Rluc activity was used as an internal control.

### qRT-PCR

Thirty-two hours after transfection, total RNA was isolated from the samples using TRIzol reagent (Invitrogen, USA) according to the manufacturer's instructions. DNA was removed from the isolated RNA using DNaseI (Takara, Japan), and then cDNA was produced using M-MLV reverse transcriptase (Promega, USA) and random hexamers. qRT-PCR was conducted in a 20-μl reaction system that consisted of 10 μl of SYBR Green PCR mix (Takara, Japan), 1 μl of cDNA (100 ng), 0.4 μM qFluc-F primer (5′-TTCGGTTGGCAGAAGCTATG-3′), and 0.4 μM qFluc-R primer (5′-GGTAGGCTGCGAAATGCCCA-3′). The GAPDH gene was assessed as an internal control using primers qGAPDH-F (5′-AGGGCTGCTTTTAACTCTGGTAAA-3′) and qGAPDH-R (5′-CATATTGGAACATGTAAACCATGTAGTTG-3′). Each qRT-PCR was conducted with three biological replicates. The amplification was conducted using a Mastercycler ep realplex real-time PCR system (Eppendorf, Germany) with the following program: 95°C for 10 min, followed by 40 cycles of 95°C for 15 s, 55°C for 45 s, and 72°C for 45 s. RNase-free water and cDNA from un-transfected RK13 cells were used as blank and negative controls, respectively. False-positive qRT-PCR results caused by primer dimers or nonspecific amplicons were eliminated from the final analysis based on a melting curve analysis of 95°C for 15 s, 60°C for 30 s, and 95°C for 15 s. In a preliminary test, the PCR programs were optimized for single bands of the expected sizes of specific genes in a 1% agarose gel. The relative expression level of the *Fluc* gene compared with the control (pRHDV-luc) was analyzed based on the comparative threshold cycle (C_T_). Briefly, the Fluc C_T_ value was normalized according to the formula, ΔC = C_T(Fluc)_−C_T(GAPDH)_, and the relative expression levels of the genes were determined using the formula, ΔΔ_CT_ = Δ_CT(sample)_ –Δ_CT(control)_. The relative expression of *Fluc* was determined according to the 2^– ΔΔct^ method.

### Mammalian two-hybrid assay

The interaction between eIF4E and RHDV VPg protein in vivo was evaluated using a CheckMate Mammalian Two-Hybrid System (Promega, USA). The protein of interest is expressed as a fusion with the GAL4 DNA-binding domain, and another protein is expressed as a fusion with the activation domain of the VP16 protein of the herpes simplex virus [20]. Briefly, the yeast GAL4 DNA-binding domain and the VPg protein were expressed using the pACT-VPg plasmid, and the pBIND-eIF4E plasmid was used to express the herpes simplex virus VP16 activation domain and eIF4E. The pG5luc vector contains five GAL4 binding sites upstream of the TATA box, which controls *Fluc* expression. The pGL4.75 vector encodes the luciferase reporter gene *Renilla reniformis* (*Rluc*) from a CMV promoter. The pACT-VPg and pBIND-eIF4E plasmids and the pG5luc vector were then transfected into RK-13 cells. Forty-eight hours after transfection, the RK-13 cells were lysed, and the expression levels of *Fluc* and *Rluc* were evaluated using the Dual-Luciferase Reporter Assay System (Promega, USA).

### Co-immunoprecipitation (Co-IP) assay

RK-13 cells were co-transfected with the pV5-VPg and pMyc-eIF4E plasmids. Forty-eight hours after transfection, total protein was isolated from PK-13 cells using IP lysis buffer. Co-immunoprecipitation (Co-IP) was conducted using a Co-IP Kit (Pierce, USA) according to the manufacturer's instructions. AminoLink Plus Coupling Resin was incubated with anti-V5 monoclonal antibody (MAb) (sc-81594; Santa Cruz) and subjected to sodium dodecyl sulfate polyacrylamide gel electrophoresis (SDS-PAGE). Immunoblot analysis of the proteins was subsequently conducted using anti-myc MAb (sc-40; Santa Cruz) and anti-V5 MAb (sc-81594; Santa Cruz).

### Bimolecular fluorescence complementation (BiFC) assay

The interaction and modification of proteins in living cells can be visualized using the bimolecular fluorescence complementation (BiFC) assay, in which non-fluorescent fragments of a fluorescent protein are fused to putative interaction partners. With high sensitivity, fine spatial resolution, and minimal perturbation of the cells, BiFC enables the observation of protein interactions through detection of the intrinsic fluorescence of the protein complex [21, 22]. RK-13 cells were grown in a 35-mm plate and transiently co-transfected with plasmids pVPg-VN (1 μg) and peIF4E-VC (1 μg), pbJunVN155 (1 μg) and pbFosVC155 (1 μg), pbJunVN155 (1 μg) and pbFos(ΔZIP)VC155 (1 μg), or pVN155(I152L) vector (1 μg) and pVC155 vector (1 μg). Twenty-four hours after transfection, the protein complexes were visualized under a fluorescence microscope with an appropriate objective, and the results were normalized by flow cytometry [23].

### eIF4E siRNA-based functional assay

The 293T cells were seeded into a 35-mm plate (NEST) at a density of 2×10^5^ cells/well. The cells were grown overnight and then transfected with eIF4E small interfering RNAs (siRNAs) (sc-35284; Santa Cruz) (0 pmol, 20 pmol, 40 pmol, 60 pmol, 80 pmol, respectively) using Lipofectamine 2000 (Invitrogen Inc. USA) according to the manufacturer's instructions. Sixteen hours after transfection, cells that were transfected with eIF4E siRNAs were co-transfected with the plasmids pRHDV-luc (2 μg), peGFP (2 μg) and pIRES-Rluc (0.5 μg) using Lipofectamine 2000 (Invitrogen Inc. USA). Forty-eight hours after transfection, Western blot analysis was used to analyze the expression of eIF4E, Rluc and eGFP, in addition, The expression levels of Rluc and Fluc were evaluated using the Dual-Luciferase Reporter Assay System (Promega, USA).

### Translation assays to assess the effect of 4EBP1 on RHDV

RK-13 cells were seeded into a 35-mm plate (NEST) (2×10^5^ cells/well). RK-13 cells grown overnight were co-transfected with p4EBP1 (0 μg, 0.3 μg, 0.6 μg, 1.0 μg, respectively), pRHDV-luc (1 μg), peGFP (0.5 μg) and pIRES-Rluc (0.5 μg) plasmids using X-tremeGENE HP DNA transfection reagent (Roche, Switzerland). Forty-eight hours after transfection, the expression of 4EBP1, eGFP and Rluc in p4EBP1-transfected cells was analyzed by Western blotting. The expression levels of Rluc and Fluc were evaluated using the Dual-Luciferase Reporter Assay System (Promega, USA).

### Statistical analyses

Statistical analyses were conducted by applying the Student’s *t* test and one-way analysis of variance (ANOVA) using the SAS 9.1 package. *P* <0.05 was considered as significantly different (*), and *P* <0.01 was considered as extremely significant different (**).

## Results

### The extreme 5′ terminal sequence of RHDV RNA is linked to VPg

Based on the genomic sequences of the strains of other genera in the *Caliciviridae* family, we speculated that the RHDV genome is linked to VPg (Fig 1A). However, the formation of VPg-linked RHDV RNA was not completely clear. It is important to note that we used an *in vitro* transcriptional system to harvest sufficient RHDV 5′ UTR for an RNA electrophoretic mobility shift assay (EMSA). To determine whether the 5′ -Extreme RNA of RHDV interacts with VPg protein, the 5′ -Extreme RNA was biotinylated at the 3′ end using the RNA 3′ End Biotinylation Kit (Pierce, USA). Biotinylated 5′ -Extreme RNA was then incubated with His-tagged VPg protein that was generated in our previous study [24]. As shown in Fig 1B, the 5′ -Extreme RNA of RHDV interacted with VPg protein, as evidenced by the observation that the VPg protein bound only to 5′ -Extreme RNA (containing a 5′ UTR) and negative control RNA (RHDV NSP2 RNA without a 5′ UTR) did not interact with VPg protein.

### VPg protein is essential for RHDV translation

**Fig 2:**
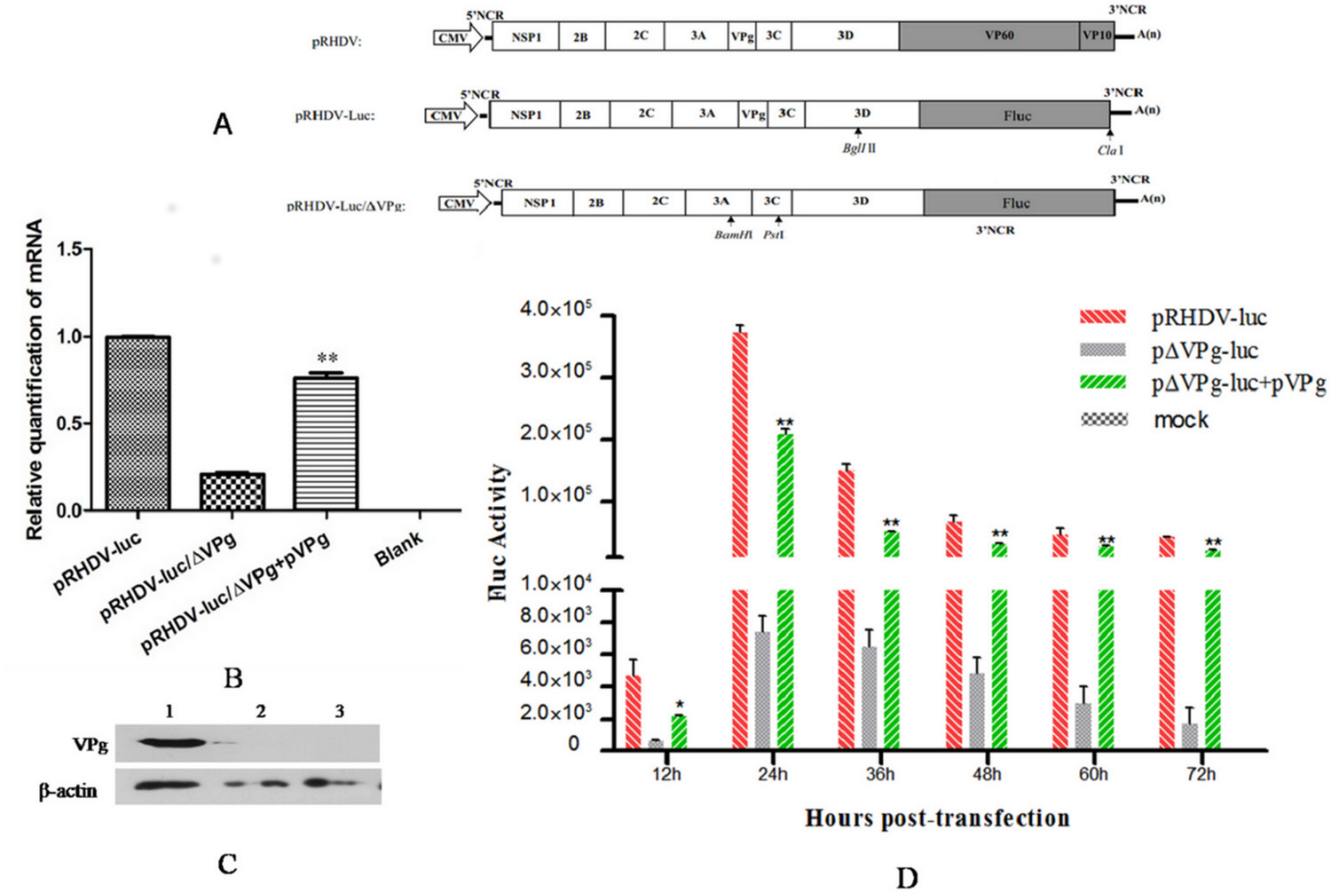
RHDV VPg protein is essential for RHDV translation. **(A)** Schematic diagram of the pRHDV-luc, pRHDV-luc/ΔVPg, and pRHDV plasmids [16]. The coding regions of viral structural proteins were replaced with Fluc using fusion PCR. In the fusion PCR results, a gray box denotes the coding region of the viral structural proteins, and lines indicate the 5′ and 3′ UTRs. **(B)** Thirty-two hours after transfection, the luciferase mRNA levels in cells transfected with pRHDV-luc, pRHDV-luc/ΔVPg + pVPg, or pRHDV-luc/ΔVPg were evaluated by qRT-PCR. The Student’s *t* test and ANOVA were applied for the statistical analyses, *P* <0.05 was considered as significantly different (*), and *P* <0.01 was considered as extremely significant different (**). **(C)** The expression level of trans-supplemented VPg was evaluated by immunoblotting with VPg polyclonal antibodies. Line 1: pVPg; line 2: pcDNA3.1 vector; line 3: blank control. **(D)** Relative luciferase activity in RK13 cells carrying pRHDV-luc/ΔVPg, trans-supplemented pVPg, and the parental genotype pRHDV-luc at 12 h, 24 h, 36 h, 48 h, 60 h, and 72 h post-transfection. The luciferase activity in RK13 cells was evaluated by measuring the firefly luciferase activity at different time points after transfection. Renilla luciferase activity measured at the same time points was used to normalize the transfection efficiency. The differences in luciferase activities associated with pRHDV-luc/ΔVPg + pVPg and pRHDV-luc/ΔVPg were compared using SAS 9.1 software. The Student’s *t* test and ANOVA were used for the statistical analyses. *P* <0.05 was considered as significantly different (*), and *P* <0.01 was considered as extremely significant different (**).The experiments were conducted in triplicate, and similar results were obtained from three independent experiments.

To explore the role of VPg in viral translation, an RHDV replicon system, pRHDV-luc [16], was used in the present study. The luciferase activity of RK13 cells transfected with pRHDV-luc, pRHDV-luc/ΔVPg, pRHDV-luc/ΔVPg+pVPg, or pGL4.75 (Rluc), was analyzed and compared. The stability of Fluc mRNA in the absence or presence of VPg was evaluated by qRT-PCR. Our results showed that the levels of Fluc mRNA derived from pRHDV-luc/ΔVPg and pRHDV-luc/ΔVPg + pVPg were approximately 20% and 80%, respectively, of that of pRHDV-luc (Fig 2B). The expression of VPg was evaluated by immunoblotting with VPg polyclonal antibodies (Fig 2C). As shown in Fig 2D, the deletion of VPg significantly reduced the expression of Fluc, and trans-complementation of VPg restored Fluc expression, suggesting that VPg is essential for viral transcription and translation.

### RHDV VPg protein interacts with eIF4E

**Fig 3:**
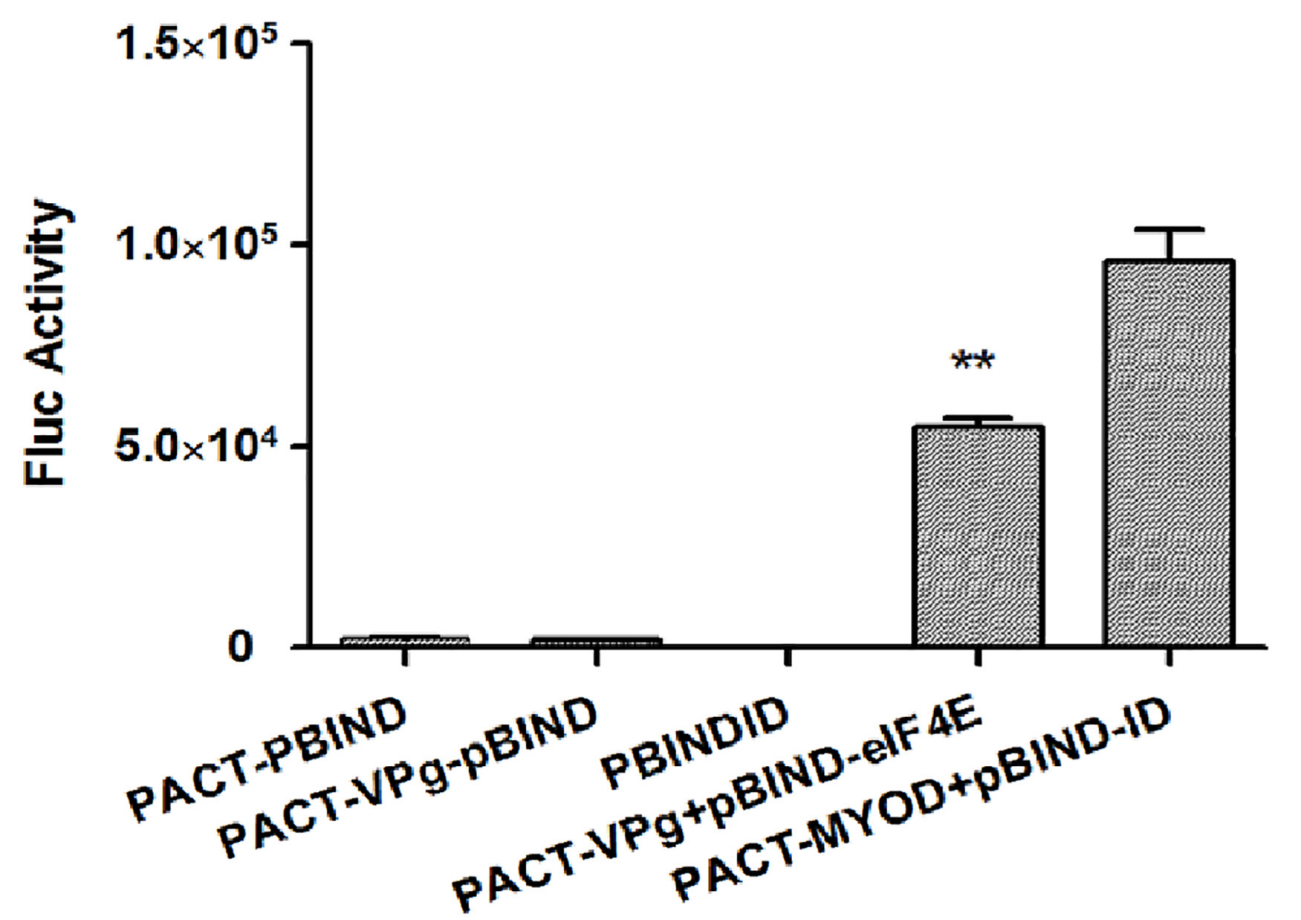
The interaction between VPg and eIF4E in RK13 cells based on the mammalian two-hybrid assay. RK13 cells (2×10^5^ cells per well in a 6-well plate) were co-transfected with the pACT (1 μg) and pBIND (1 μg) plasmids, pACT-VPg (1 μg) and pBIND (1 μg) plasmids, and pACT-VPg (1 μg) and pBIND-eIF4E (1 μg) plasmids, respectively. RK13 cells co-transfected with the pACT-MyoD (1 μg) and pBIND-ID (1 μg) plasmids served as positive controls [25]. A blank control was also used. In addition, all of the groups were transfected with pGL4.75 (0.2 μg). The cells were lysed at 48 h post-transfection, and Fluc activity was evaluated based on RLUs and normalized according to the results obtained for a co-transfected plasmid encoding the Renilla luciferase. The experiments were conducted in triplicate, and similar results were obtained from three independent experiments. The Student’s *t* test and ANOVA were applied for the statistical analyses, *P* <0.01 was considered as extremely significant different (**).

While it has been reported that feline calicivirus (FCV) and murine norovirus (MNV) VPg interact with eIF4E [14], it is not known whether RHDV VPg interacts with eIF4E. In the present study, we used the mammalian two-hybrid assay, a powerful tool for the detection of protein interactions *in vivo*, to evaluate the potential interaction between VPg and eIF4E in RK-13 cells. In the mammalian two-hybrid assay, the transcriptional activation and DNA-binding domains, which are produced by distinct plasmids, are closely associated if one protein that is fused to a transcriptional activation domain interacts with another protein that is fused with a DNA-binding domain [20]. As shown in Fig 3, the Fluc activities of VPg-eIF4E and the positive control were significantly higher than the activity in the control group, suggesting that VPg interacted with eIF4E.

**Fig 4:**
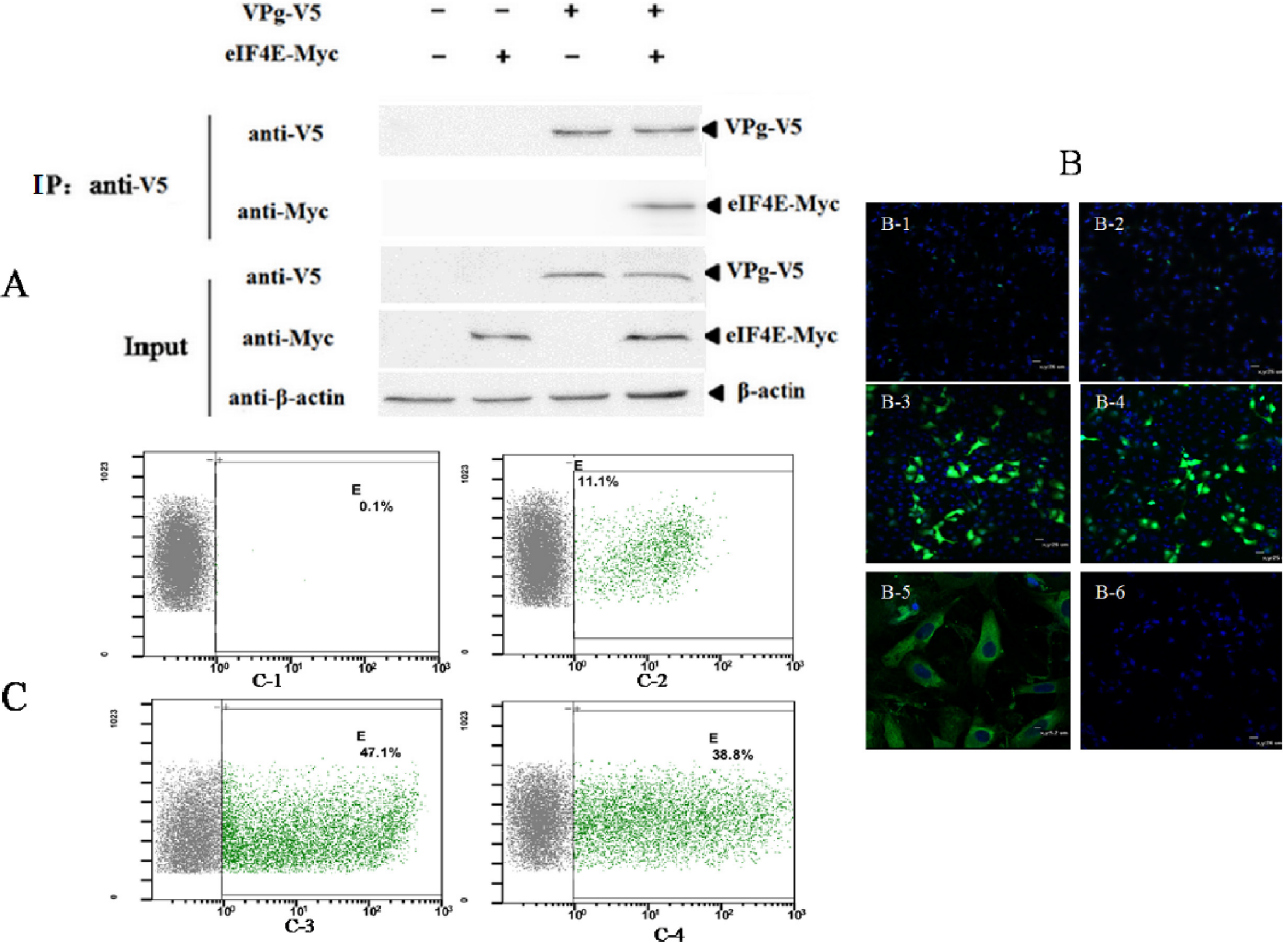
The interaction between RHDV VPg protein and eIF4E. **(A)** Co-IP of VPg and eIF4E protein. RK13 cells were co-transfected with the indicated plasmids (+) or empty vectors (-). Next, whole-cell lysates obtained at 48 h post-transfection were immunoprecipitated (IP) using anti-V5 MAb. The proteins were separated by SDS-PAGE and detected by immunoblotting with specific antibodies. The protein identities are shown below the panel. **(B)** BiFC of VPg and eIF4E protein. RK-13 cells grown in a 35-mm plate were transiently co-transfected with vectors pVN155(I152L) (1 μg) and pVC155 (1 μg) (B-1), negative control vectors pbJunVN155 (1 μg) and pbFos(ΔZIP)VC155 (1 μg) (B-2), a combination of plasmids pVPg-VN (1 μg) and peIF4E-VC (1 μg) (B-3), the positive control pbJunVN155 and pbFosVC155 (Jun-Fos is a heterodimer[26]) (B-4), the VPg and eIF4E subcellular localization interaction (B-5), and the blank control (B-6). At 24 h post-transfection, the BiFC complexes were visualized under a fluorescence microscope with an appropriate objective. The position of the nuclei was determined using DAPI (blue). **(C)** BiFC of VPg and eIF4E proteins by flow cytometry. During the BiFC experiments, the green fluorescence observed at 48 h post-transfection was quantified by flow cytometry. C-1: pVN155(I152L) and pVC155 vectors; C-2: negative control pbJunVN155 and pbFos(ΔZIP)VC155; C-3: positive control pbJunVN155 and pbFosVC155; C-4: combination of plasmids pVPg-VN and peIF4E-VC. The gray cloud means negative cells which have no green fluorescence; the green cloud means positive cells which have green fluorescence; X axes represents the fluorescence intensity; Y axes represents cell size; 'E' represents the ratio of the positive cells to the negative cells.

To confirm the interaction between VPg and eIF4E, a Co-IP experiment was conducted in RK-13 cells transiently co-expressing V5-tagged VPg and Myc-tagged eIF4E. Cells co-expressing Myc and V5-tagged VPg were used as controls. Co-IP using anti-V5 MAb revealed that V5-VPg formed a complex with Myc-eIF4E rather than Myc (Fig 4A). To visualize the interaction between VPg and eIF4E, a bimolecular fluorescence complementation (BiFC) assay was conducted in RK-13 cells. As shown in Fig 4B, the VPg-eIF4E interaction was observed in cytoplasm of RK-13 cells. Quantitative analysis of the green fluorescence by flow cytometry showed that the BiFC signal-to-noise (Ratio between the treatment and control groups) was greater than three at 48 h post-transfection (Fig 4C). Therefore, those results suggested that VPg interact with eIF4E.

### RHDV translation is sensitive to the VPg-eIF4E interaction

**Fig 5:**
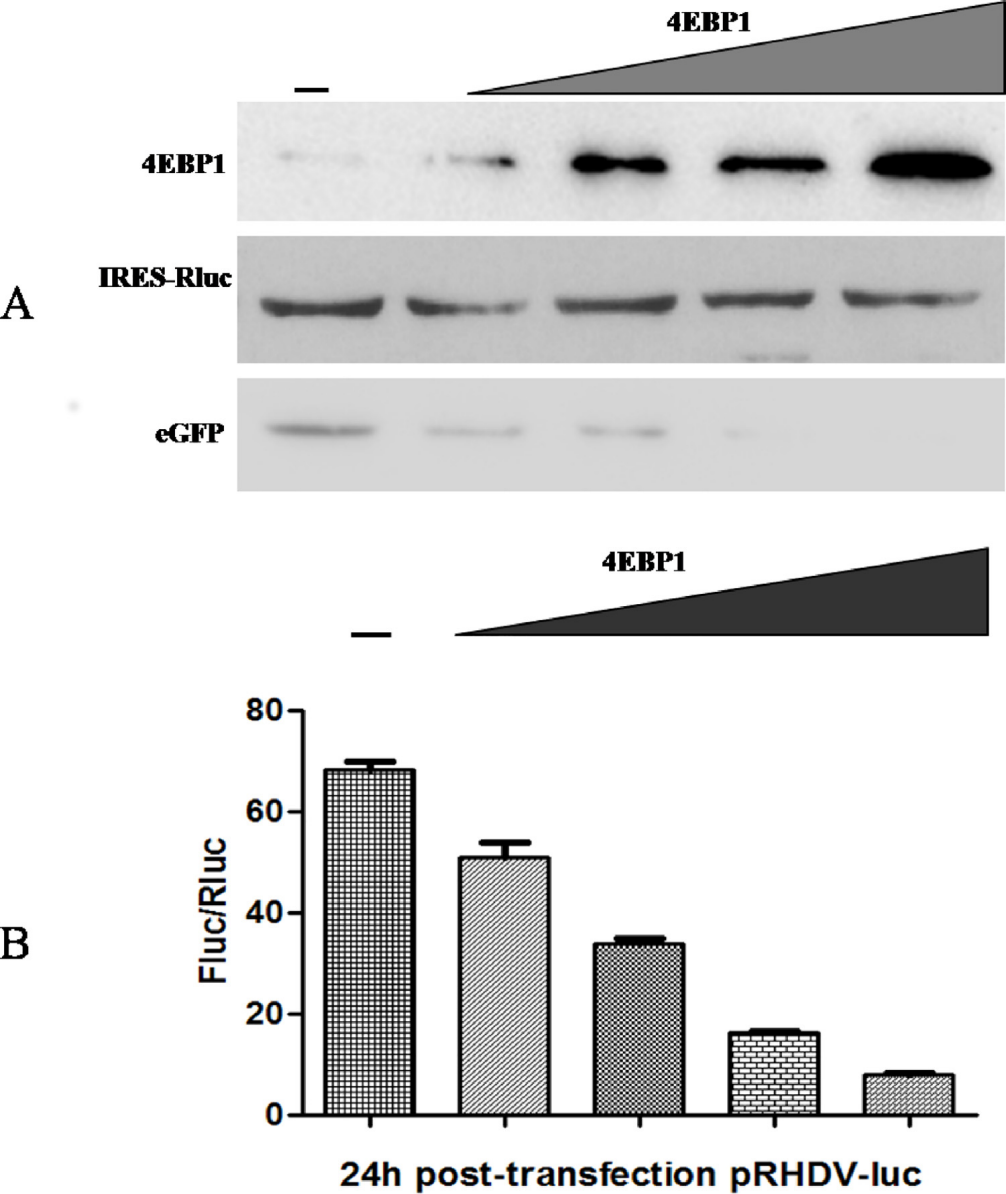
Knockdown of VPg and eIF4E protein interactions decreased the levels of RHDV translation. RK13 cells were co-transfected with the p4EBP1 (0 μg, 0.3 μg, 0.6 μg, or 1.0 μg), pRHDV-luc (1 μg), peGFP (0.5μg) and pIRES-Rluc (0.5 μg) plasmids. (**A)** RK13 cells were lysed at 48 h post-transfection, and the levels of 4EBP1, eGFP and Rluc were determined by immunoblotting with antibodies against the indicated proteins. (**B)** RK13 cells were lysed at 48 h post-transfection, and Fluc activity was measured based on RLUs and normalized according to the results obtained for a co-transfected plasmid encoding Renilla luciferase. The experiments were conducted in duplicate, and similar results were obtained in two independent experiments.

To further explore the role of the VPg-eIF4E interaction in RHDV translation, we investigated the effects of eIF4E depletion on RHDV translation. As 4EBP1 protein is a specific binding protein of eIF4E, we chose it as a competitor to inhibit the binding ability of VPg to eIF4E. RK-13 cells were co-transfected with the p4EBP1 (using gradually increasing doses), pRHDV-luc and peGFP plasmids together with the pIRES-Rluc plasmid. Forty-eight hours after transfection, the RK-13 cells were lysed, and the levels of Rluc and Fluc were quantified using a Dual-Luciferase Reporter Assay System (Promega, USA). As shown in Fig 5, the translation of Fluc and eGFP decreased with increasing doses of p4EBP1 plasmid, suggesting that RHDV translation is sensitive to the VPg-eIF4E interaction.

### eIF4E is essential for RHDV replication and translation

**Fig 6:**
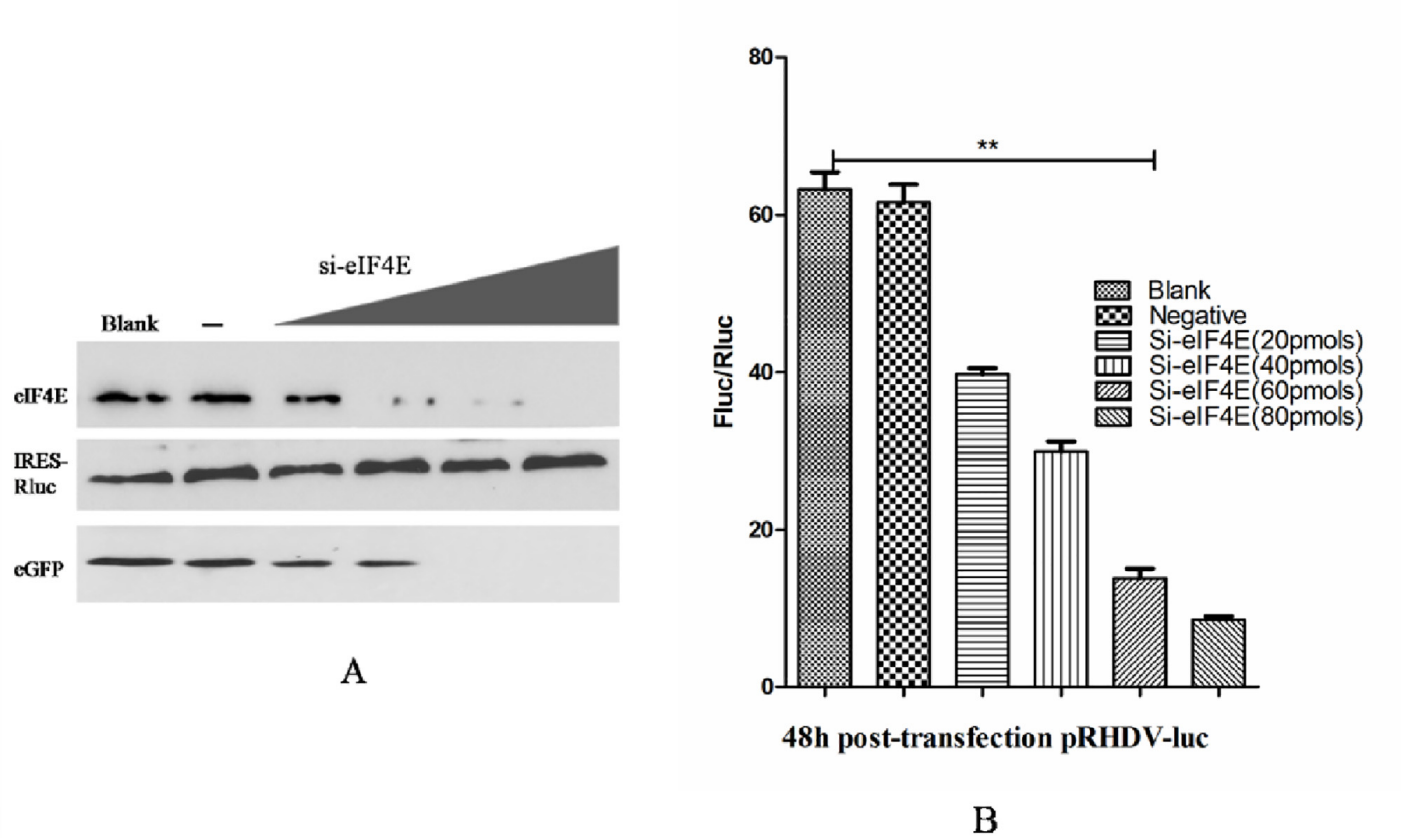
Silencing of eIF4E inhibited RHDV translation. (A) The expression of eIF4E was silenced by siRNA. The 293T cells transfected without siRNA (Mock), eIF4E siRNA (si-eIF4E), untreated (No treat), or treated with different concentrations (nM) of si-eIF4E (as indicated at the top of each lane) were harvested at 48 h post-transfection. The expression of eIF4E was measured by immunoblotting with antibodies against the indicated proteins. (B) The translation of RHDV in eIF4E-silenced siRNA, pRHDV-luc and peGFP plasmids co-transfected cells were conducted as described in panel A. The cells were lysed at 48 h post-transfection, and Fluc activity was measured based on RLUs and normalized according to the results obtained for a co-transfected pIRES-Rluc plasmid encoding Renilla luciferase. The experiments were conducted in duplicate, and similar results were obtained from two independent experiments. The Student’s *t* test and ANOVA were applied for the statistical analyses, *P* <0.01 was considered as extremely significant different (**).

To understand the role of eIF4E in RHDV replication and translation, we silenced the expression of eIF4E by using a siRNA approach. Significantly reduced expression of eIF4E was confirmed following the transfection of increasing levels of eIF4E siRNA (Fig 6A). Subsequently, co-transfection of the pRHDV-luc and peGFP plasmids together with the pIRES-Rluc plasmid demonstrated an eIF4E siRNA-mediated inhibition of viral replication and translation (Fig 6B), suggesting that eIF4E is essential for RHDV replication and translation.

## Discussion

In general, two mechanisms are involved in translation initiation in positive-strand RNA viruses. The 5′ cap-dependent mechanism contributes to translation initiation in *Alphaviruses*, *Flaviviruses*, *Coronaviruses*, and *Arteritis viruses*, whereas viruses such as *Rhinovirus*, *Enterovirus Cardiovirus*, and *Aphthovirus* utilize a cap-independent strategy for translation initiation. In the cap-independent strategy, ribosomes bind directly to an internal site of the 5′ non-coding region (NCR) of the viral genome. The 5′ UTR of caliciviruses is significantly shorter than that of other positive-stranded RNA virus genomes. For example, the RHDV 5′ UTR comprises only 9 nt. In addition, there is no cap structure at the 5′ end of *Calicivirus* genomic RNA. Thus, a novel mechanism is involved in the translation initiation of caliciviruses.

Goodfellow *et al*. demonstrated that feline calicivirus (FCV) and murine norovirus (MNV) use a novel protein-directed translation initiation mechanism [13, 14, 27], in which the translation initiation factors interact with VPg. This novel translation initiation mechanism has been discovered in some plant viruses, for example, potyvirus translation initiated by VPg interact with eukaryotic initiation factor (eIF) iso 4E (eIF(iso)4E) [28] and turnip mosaic virus (TuMV) translation initiation directed by VPg forms a ternary complex with eIF(iso)4E and eukaryotic initiation factor (eIF) iso 4G (eIF(iso)4G) [29, 30]. But it has never been reported in other animal RNA viruses. RHDV is one of the most important members of the Lagovirus genera in the *Caliciviridae* family. The molecular mechanisms underlying RHDV translation and replication are largely unknown due to the lack of suitable tissue culture systems. We recently established an experimental RHDV replicon system [16], in which a Fluc gene is fused in-frame with the viral ORF of the deleted structural genes of the virus. The translation and replication levels of the replicon can be evaluated by detecting a reporter gene. In the present study, to elucidate the role of VPg in RHDV translation, we deleted the coding region of VPg in the replicon. Transfected RK-13 cells revealed that the VPg deletion significantly reduced the expression of the reporter gene, suggesting that VPg was involved in viral translation. The expression level of Fluc was almost completely restored when VPg was trans-supplemented by transfecting the replicon into RK-VPg cells, suggesting that VPg plays an important role in the life cycle of RHDV.

To date, only norovirus VPg has been shown to interact with eIF3 and eIF4E; however, an in vitro study of a rabbit reticulocyte lysate revealed that eIF4E is not essential for the translation of MNV RNA [13]. Interestingly, the interaction between VPg and eIF4E is necessary for translation initiation of FCV mRNA. Therefore, it is likely that VPg interacts with multiple components of the translation initiation factor complex and that not all of these interactions are essential for viral RNA translation. In the present study, we found that RHDV VPg interacted with eIF4E, and both VPg and eIF4E were essential for the translation of RHDV in RK-13 cells. These findings suggest that RHDV VPg also acts as a proteinaceous “cap substitute” to attract ribosomes to the viral mRNA through interactions with the cap-binding protein eIF4E. Further functional studies of VPg and RHDV translation are ongoing to determine whether all of the components of the eIF4F complex are required for the initiation of translation.
